# Multifunctional Evaluation of Graphene Oxide–Sulfonamide Nanoconjugates: Antimicrobial, Antibiofilm, Cytocompatibility and Xenobiotic Metabolism Gene Expression Insight

**DOI:** 10.3390/molecules30122585

**Published:** 2025-06-13

**Authors:** Irina Zarafu, Irina Mușat, Carmen Limban, Diana C. Nuță, Ioana Daniela Dulama, Cristiana Radulescu, Raluca Maria Stirbescu, Arnaud Tatibouet, Carmen M. Chifiriuc, Luminita Marutescu, Marcela Popa, Laura D. Dragu, Elena Radu, Ioana Nicolau, Coralia Bleotu, Petre Ionita

**Affiliations:** 1 Faculty of Chemistry, University of Bucharest, 90 Panduri, 050663 Bucharest, Romania; ana.musat@s.unibuc.ro (I.M.); ioana.nicolau@chimie.unibuc.ro (I.N.); 2 Research Institute of the University of Bucharest (ICUB), University of Bucharest, 060023 Bucharest, Romania; carmen.chifiriuc@bio.unibuc.ro (C.M.C.); luminita.marutescu@bio.unibuc.ro (L.M.); marcela.popa@bio.unibuc.ro (M.P.); cbleotu@yahoo.com (C.B.); 3 Faculty of Pharmacy, “Carol Davila” University of Medicine and Pharmacy, 6 Traian Vuia, 020956 Bucharest, Romania; carmen.limban@umfcd.ro (C.L.); diana.nuta@umfcd.ro (D.C.N.); elena.radu@virology.ro (E.R.); 4 Institute of Multidisciplinary Research for Science and Technology, Valahia University of Targoviste, 130004 Targoviste, Romania; dulama.ioana@icstm.ro (I.D.D.); stirbescu.raluca@icstm.ro (R.M.S.); 5 Faculty of Sciences and Arts, Valahia University of Targoviste, 130004 Targoviste, Romania; cristiana.radulescu@valahia.ro; 6 Doctoral School Chemical Engineering and Biotechnology, National University of Science and Technology Politehnica of Bucharest, 060042 Bucharest, Romania; 7 Academy of Romanian Scientists, 050044 Bucharest, Romania; 8 Institut de Chimie Organique et Analytique (ICOA), Université d’Orléans-CNRS FR 2708-UMR 7311, 45067 Orléans, France; arnaud.tatibouet@univ-orleans.fr; 9 Faculty of Biology, University of Bucharest, 91-95 Spl. Independentei, 050095 Bucharest, Romania; 10 Ştefan S Nicolau Institute of Virology, Romanian Academy, 030304 Bucharest, Romania; denisa_dragu81@yahoo.com

**Keywords:** graphene oxide, sulfonamide, antimicrobial, apoptosis, cell cycle, cytochrome P450, N-acetyltransferase expression

## Abstract

The clinical utility of sulfonamide antibiotics is increasingly challenged by antimicrobial resistance and pharmacokinetic limitations. In this study, we synthesized five graphene oxide–sulfonamide nanoconjugates (GO–S1 to GO–S5) via covalent functionalization, comprehensively characterized them by IR, Raman, SEM, EDS, etc., and evaluated their antimicrobial, antibiofilm, cytotoxic, apoptotic, hemolytic and gene expression-modulating effects. While the free sulfonamides (S1–S5) exhibited superior antimicrobial activity in planktonic cultures (MICs as low as 19 μg/mL), their GO-functionalized counterparts demonstrated enhanced antibiofilm efficacy, particularly against *Pseudomonas aeruginosa* (MBIC: 78–312 μg/mL). Cytotoxicity studies using CellTiter assays and Incucyte live-cell imaging revealed low toxicity for all compounds below 250 μg/mL. Morphological and gene expression analyses indicated mild pro-apoptotic effects, predominantly via caspase-9 and caspase-7 activation, with minimal caspase-3 involvement. Hemolysis assays confirmed the improved blood compatibility of GO–Sx conjugates compared to GO alone. Furthermore, qRT-PCR analysis showed that GO–Sx modulated the expression of key xenobiotic metabolism genes (CYPs and NATs), highlighting potential pharmacokinetic implications. Among all tested formulations, GOS3, GOS4 and GOS5 emerged as the most promising candidates, balancing low cytotoxicity, high hemocompatibility and strong antibiofilm activity. These findings support the use of graphene oxide nanocarriers to enhance the therapeutic potential of sulfonamides, particularly in the context of biofilm-associated infections.

## 1. Introduction

Antimicrobial resistance (AMR) ranks among the top ten global health threats, impacting not only human populations but also animals and the environment. Antibiotics exert their effects through either bactericidal mechanism—destroying bacteria—or bacteriostatic mechanisms—inhibiting bacterial growth and reproduction.

Sulfonamides (often called “sulfa drugs”) are antimetabolite antibiotics that disrupt bacterial folate synthesis, halting the production of DNA, thereby inhibiting bacterial growth and cell division [1,2].

Notably, sulfonamides exhibit a broad spectrum of activity against many Gram-positive and Gram-negative bacteria. Their broad tissue distribution and oral bioavailability made them pioneering systemic antibiotics [3]. Another advantage is that sulfonamides selectively target bacterial folate synthesis—human cells are unaffected because we obtain folate from our diet rather than synthesizing it de novo [2]. This selective mechanism contributed to the early success of sulfonamides in treating infections while sparing host cells. Sulfonamides continue to be useful in clinical settings, even as many bacteria have developed resistance (often via acquired *sul* genes encoding drug-resistant synthase enzymes, or mutations that increase PABA production), due to their unique niche applications and the lack of complete replacements for certain conditions [2]. Although sulfonamides are no longer first-line for many routine infections, they remain nonetheless indispensable for specific infections and patient populations, often as part of combination therapies or when other options are limited [4]. Key clinical applications that maintain sulfonamides’ relevance include *Pneumocystis jirovecii* pneumonia (PCP) in immunocompromised patients, such as people with HIV/AIDS or other immunosuppression, acute *Toxoplasma gondii* infections (e.g., toxoplasmic encephalitis) and toxoplasmosis prophylaxis in high-risk patients, *Nocardia* pulmonary or disseminated infections [4,5], urinary tract infections when pathogens are known to be susceptible or when first-line agents (like nitrofurantoin or fluoroquinolones) cannot be used, skin and soft tissue infections (including community-acquired methicillin-resistant *S. aureus* infections [6] and particular *protozoal infections* (e.g., *Isospora* diarrhea). In veterinary medicine, sulfonamides are still widely used for livestock and poultry infections (which unfortunately has contributed to environmental resistance genes) [4]. Additionally, sulfonamide–antibiotic combinations have shown activity in experimental settings (e.g., sulfonamide plus an antifungal azole exhibited synergy against fungal pathogens) [7].

Despite their historical significance and broad utility, sulfonamides—like many conventional antibiotics—face increasing resistance from bacterial strains. The emergence and spread of such resistance present ongoing clinical challenges and underscore the urgent need for innovative strategies to enhance the efficacy of existing antimicrobials.

In this context, our study explores the potential of nanoparticle conjugation to improve the antimicrobial activity of sulfonamides, aiming to overcome current limitations and reinvigorate their clinical relevance.

Nanoparticles have been increasingly researched for their potential to be used in treating bacterial infections due to their unique properties in targeted drug delivery and even, in the case of some, antibacterial and antibiofilm properties. Graphene oxide (GO) is an insoluble material based on a carbon lattice with abundant functional groups, like hydroxyl, carboxyl, keto and epoxy [8]. These groups make it suitable for chemical functionalization, but physical absorption may occur. Literature data showed that sulfonamides are readily absorbed onto graphene or reduced graphene oxide [9,10], but this absorption depends on several factors, including pH. The equilibrium and thermodynamic performance and kinetic measurements of the sulfonamide adsorption onto graphene were recently studied [11].

**Figure 1:**
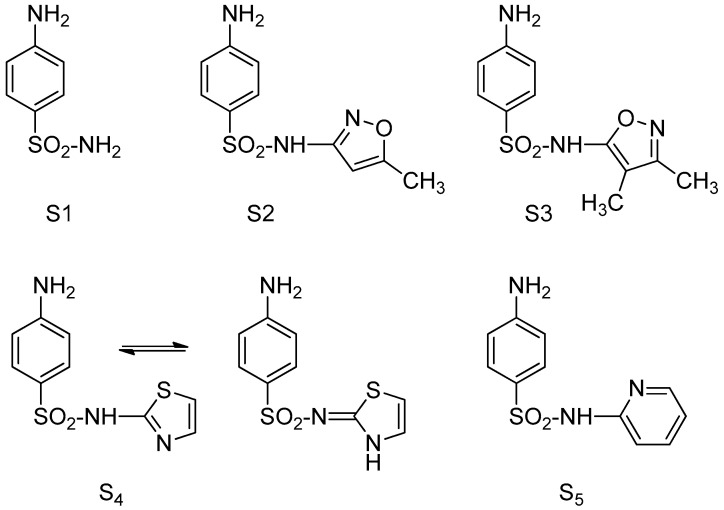
Chemical structures of the involved sulfonamides.

In our experiments we used *para*-aminobenzene sulfonamide (sulfanilamide S1), 4-amino-*N*-(5-methyl-1,2-oxazol-3-yl) benzene sulfonamide (sulfamethoxazole S2), 4-amino-*N*-(3,4-dimethyl-1,2-oxazol-5-yl) benzene sulfonamide (sulfafurazole/sulfisoxazole, S3), 4-amino-*N*-(1,3-thiazol-2-yl) benzene sulfonamide (sulfathiazole S4) with wide or restricted spectrum of activity on *Gram-positive* and *Gram-negative* bacteria (Figure 1) [12,13,14,15,16,17,18,19,20,21,22,23,24,25,26] and 2-hydroxy-5-((4-(pyridin-2-ylsulfamoyl)phenyl)diazinyl) benzoic acid (sulfasalazine S5), a sulfonamide with anti-inflammatory and immunosuppressive properties [27].

The sulfonamides were covalently linked to graphene oxide and have been further used in biological assays. We hypothesize that integrating them into graphene-based materials could significantly enhance their antibacterial potential, enabling their incorporation into various carriers such as gels, polymers or solutions.

## 2. Results

### 2.1. Chemical and Structural Characterization

**Figure 2:**
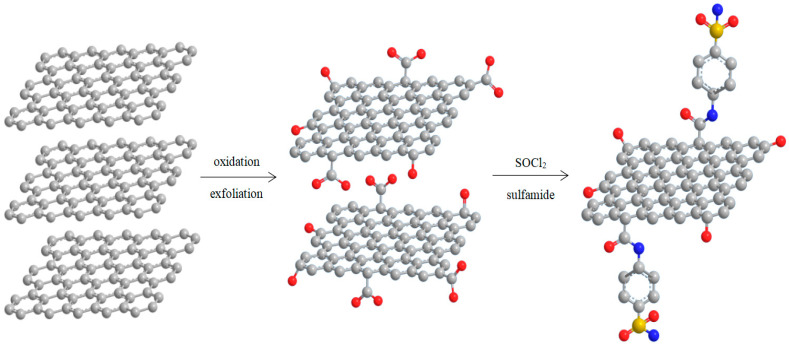
Typical synthesis of the graphene oxide functionalized with sulfonamides.

The synthesis of the GO covalently functionalized with sulfonamides was achieved using previously described procedures [28,29,30,31,32,33,34,35]. Thus, graphite was oxidized first to GO using the Hummers method [36] and then activated by thionyl chloride to covalently bind the sulfonamides containing an amino group (Figure 2).

**Figure 3:**
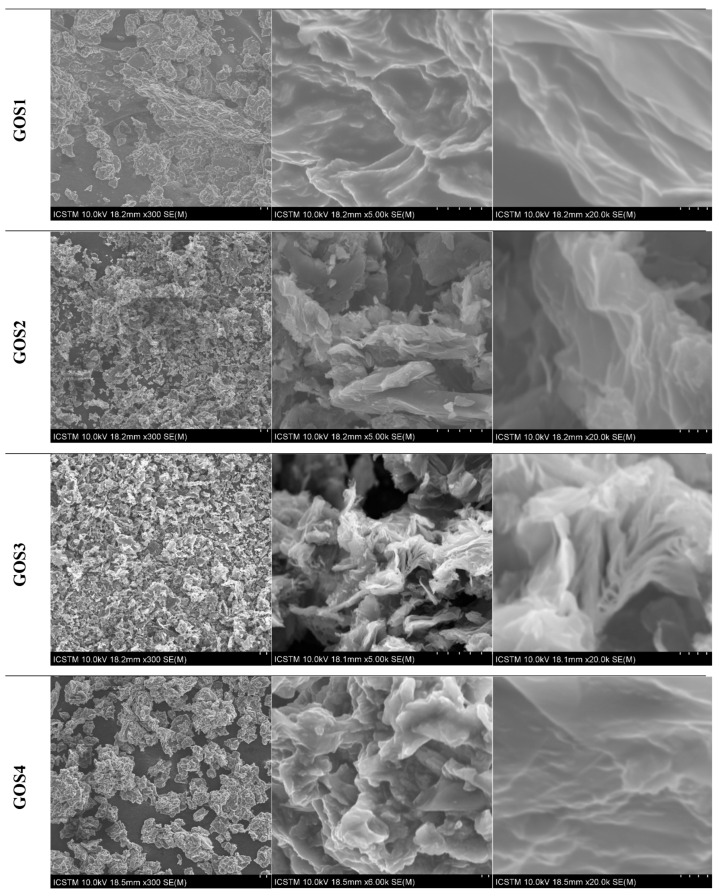

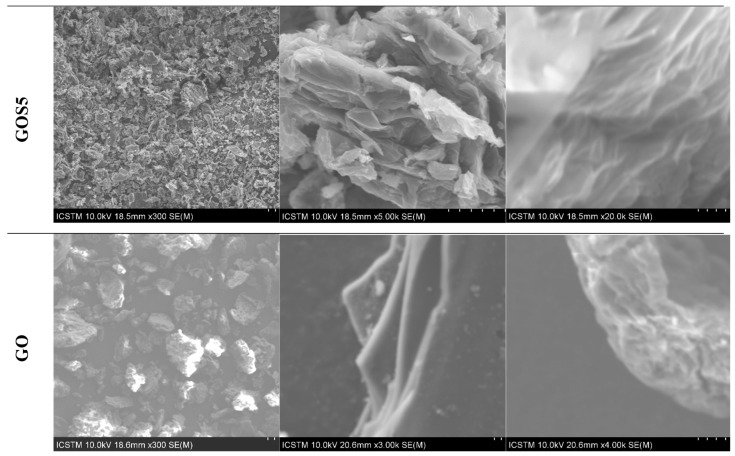
SEM images at different magnifications.

The SEM investigation on the morphology of new functionalized GO reveals that the structures of all synthesized samples differ depending on the type of sulfonamides grafted onto the GO (Figure 3). Thus, Figure 3 shows the SEM images (at different magnification order) recorded on synthesized samples; the morphological differences can be due to sample preparation techniques or the sulfonamides type used to functionalize graphene oxides. All samples present smooth surfaces with wrinkles and folded regions—characteristic of graphene materials. As can be seen in images recorded at 300×, the samples have different grain sizes and/or agglomeration modes. Comparative with the initial sample, the functionalized samples are characterized by smaller grain sizes and thinner lamellar structures—these characteristics can be easily observed in the SEM images recorded at 20 kx magnification order.

**Figure 4:**
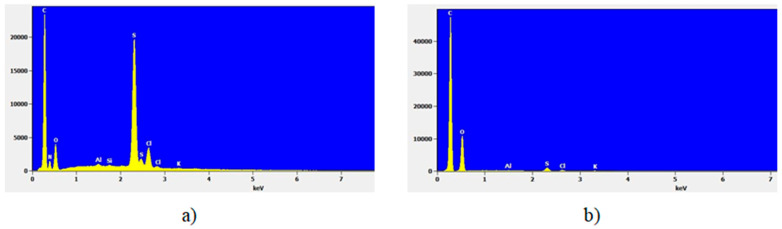
EDS spectrum of analyzed samples: (**a**) graphene oxide functionalized with sulfonamides; (**b**) initial graphene oxide.

Figure 4a shows a specific EDS spectrum of the GO-S_x_, while Figure 4b shows the EDS spectrum of the initial GO. This reveals the presence of C, N, O, S and Cl (as significant components, e.g., C_x_ > 1 wt.%) in all samples and of other elements as Na, Al, Si, K and Ca (as minor or trace components, e.g., 0.01 wt.% < C < 1 wt.%). The elemental content results are centralized in Table 1.

The results obtained by EDS reveal that the mean value of nitrogen in GO-S_x_ was 6.40 wt.% while, in the initial GO sample, this element was not detected. In a similar manner, the mean value of sulfur content in GO-S_x_ samples was 15.83 wt.% while in the initial GO sample was recorded 2.15 wt.% and the mean value of chlorine content in GO-S_x_ was 4.56 wt.% while in the initial GO sample was recorded 0.82 wt.%. All these differences appear from the synthesis process.

Vibrational spectroscopy, including ATR-FTIR and Raman scattering, enables the characterization of the chemical structure of graphene-incorporated sulfonamides derivatives. FTIR data of synthetized graphene oxide–sulfonamides derivatives are shown in Table 2.

The functional groups of graphene oxide (GO) as well as of GO-S_x_ revealed broad peaks at 3408 (GO), 3392 (GOS1), 3397 (GOS2), 3398 (GOS3), 3392 (GOS4) and 3389 (GOS5) cm^−1^, attributed to the OH stretching vibration mode, and medium peaks at 1720 (GO), 1724 (GOS1), 1714 (GOS2), 1730 (GOS3), 1715 (GOS4) and 1722 (GOS4) assigned to C=C stretching vibration modes, respectively [28,37]. The medium intensity peaks around 1404 and 1238 cm^−1^ (Table 2) are assigned to functional groups such as C–O and C–O–C, respectively. Furthermore, the normalized FTIR data of GO and GO-S_x_ revealed peaks with medium/strong intensity around 1593–1620, 1388–1394, 1217–1229 and ~1085 cm^−1^ for C=C aromatic stretch, C–O–C ether, C–OH stretch and C–O–C groups, respectively. The intensity of peaks of functionalized GO-sulfonamides groups was highlighted in the range of 1537–1539 cm^−1^ for C-N and N-H stretching vibrations. The same assignment of sulfonamide functional group can be made to medium/strong intensity peaks in the range of 3208–3322 cm^−1^ for N-H stretching vibration and medium signal in the range of 1313–1326 cm^−1^ for C-N stretching vibration mode, respectively. The weak peaks in the range of 2037–2254 cm^−1^ were assigned to C-S-N stretching vibration of functionalized sulfonamides. Additionally, the weak intensity peaks observed in FTIR spectra with an intensity between 1545 and 1566 cm^−1^ were assigned to C=C stretching vibration of *sp^2^* hybridized carbon atoms from graphene oxide structures, as well as to C-N, N-H stretching vibration mode of sulfonamides. The medium intensity peaks at 3169, 3161 and 3170 cm^−1^, were attributed to the C=O stretching vibration. However, in the FTIR spectra and data centralized in Table 2, the strong peaks around 1180 and 1142 cm^−1^ were attributed to the S=O stretching vibration mode of –SO_2_-NH- group (sulfonamides). The minimum intensity of peaks of all synthesized compounds, including GO, according to FTIR data, in the range of 3620–3709 cm^−1^ (Table 2), were assigned to NH_2_ symmetric/asymmetric group.

**Figure 5:**
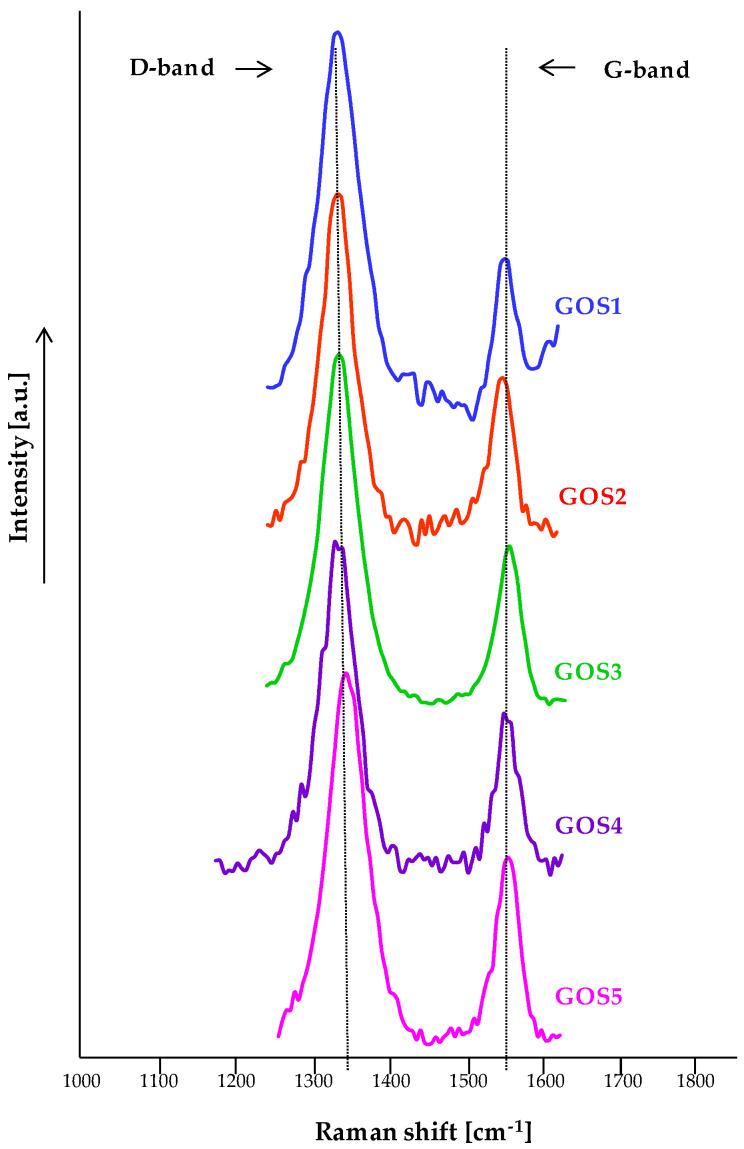
Overlap of Raman spectra for the GO-S_x_: GOS1, GO-para-aminobenzene sulfonamide; GOS2, GO-4-amino-N-(5-methyl-1,2-oxazol-3-yl) benzene sulfonamide; GOS3, 4-amino-N-(3,4-dimethyl-1,2-oxazol-5-yl) benzene sulfonamide; GOS4, GO-4-amino-N-(1,3-thiazol-2-yl) benzene sulfonamide; GOS5, GO-2-hydroxy-5-((4-(pyridin-2-ylsulfamoyl)phenyl)diazinyl) benzoic acid.

Regardless of the chemical structure, the Raman spectra of the GO-S_x_ display a few notable peculiarities. In this context, Figure 5 shows the peak positions corresponding to D and G bands of graphene; the ratio between intensity of D band and intensity of G band (I_D_/I_G_) represents the degree of structural ordering or the number of defects in the functionalized graphene oxide, while the mean crystallite size can be determined using the next equation [37]:

LGOnm=2.4×10−10λ4IDIG

where L_GO_ = the mean crystallite size (in nm), λ = wavelength of the LASER source (in nm), I_D_/I_G_ = the ratio between intensity of D band and intensity of G band.

For the analyzed samples, the I_D_/I_G_ ratios have recorded the following values: 2.478 (GOS1), 2.075 (GOS2), 2.269 (GOS3), 1.971 (GOS4) and 2.020 (GOS5), respectively. These values confirm the graphene oxide structure and suggest that sp^2^ domains were formed and that the number of defects increased. The obtained results are in good accordance with other similar studies [37]. The mean crystallite size of the functionalized graphene oxide with sulfonamides was 124.140 nm (GOS1), 148.238 nm (GOS2), 135.574 nm (GOS3), 156.092 nm (GOS4) and 152.268 nm (GOS5), respectively.

### 2.2. Biological Activity

#### 2.2.1. Antibacterial and Antibiofilm Activity

**Figure 6:**
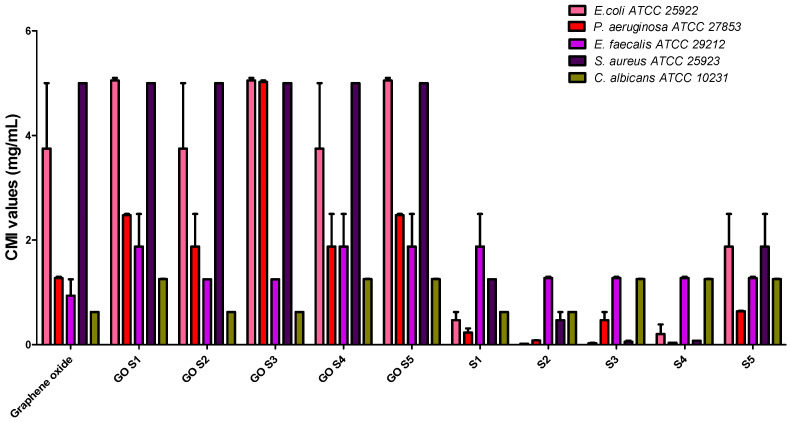
Graphic representation of the MIC values obtained for the tested S and GO-S_x_.

The GO, GOS1–GOS5 and S1–S5 exhibited antimicrobial activity against the tested *Gram-positive* and *Gram-negative* bacterial and fungal strains, with MIC values of 5–0.625 mg/mL for GO, 0.019–1.25 mg/mL for the positive controls S1–S5 and 1.25–5 mg/mL for GOS1-GOS5 (Table 3, Figure 6). The MIC values for S1–S5 indicated better antimicrobial effects against the tested strains than GO and GOS1-GOS5. The S2, S3 and S4 compounds exhibited the best antimicrobial activity against *E. coli* ATCC 25922, with MIC values of 0.019 mg/mL. None of the GO functionalized compounds did show improved antimicrobial activity, demonstrating that the pharmacologically active groups are blocked or chemically inactivated during functionalization.

**Figure 7:**
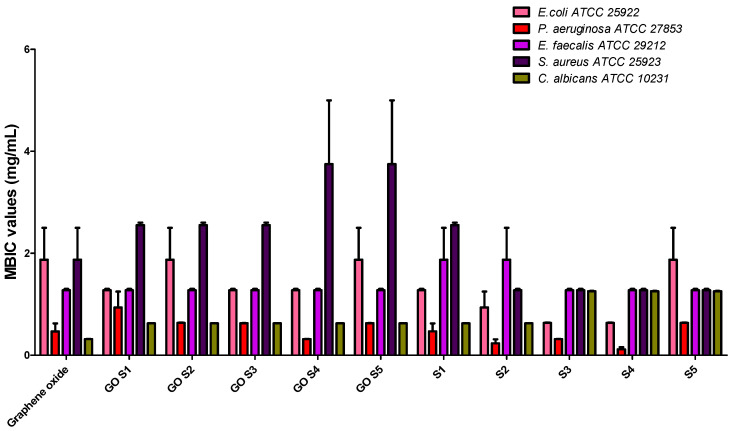
Graphic representation of the MBIC values obtained for the tested S and GO-S_x_.

The antibiofilm effects of the tested compounds and functionalized nanosystems are given in Table 4 and Figure 7. In comparison to the results obtained on planktonic microbial cells, generally, the S1–S5 compounds exhibited a similar anti-biofilm activity with that of the corresponding functionalized nanosystem, as demonstrated by the very close MBIC values. A significant result is that the GO functionalized with S3, S4 and S5 proved an improved antibiofilm activity, as revealed by the lower MBIC values. Also, it is to be noticed the very good inhibitory activity against *P. aeruginosa* ATCC 27853 biofilm development on the plastic wells of the microplates that has been prevented at very low concentrations, i.e., 0.312–0.078 mg/mL. No significant differences were observed between the tested S or GO-S_x_ regarding the anti-biofilm activity against the *Gram-positive* strains.

#### 2.2.2. Mammalian Cells’ Toxicity of S and GO-S_x_

**Figure 8:**
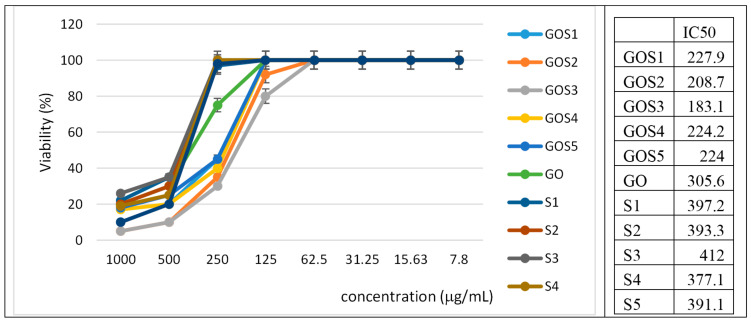
Cellular toxicity assay of S and GO-S_x_ measured by CellTiter-Glo (Promega) and reported to the untreated cells. Right: Calculated IC50.

The toxicity was evaluated using the CellTiter assay and further validated with the Incucyte live-cell imaging system—an automated, non-invasive platform that continuously monitors cells in real time. The Incucyte system captured images at regular intervals without the need to remove culture plates from the incubator or perform manual handling, enabling direct visualization of cellular responses to the tested compounds. This approach provided critical insights into cell proliferation dynamics and cytotoxic effects. Cells exposed to high concentrations of S and GO-S_x_ exhibited pronounced morphological alterations, including membrane blebbing and cell shrinkage—hallmarks of apoptosis—indicating that these compounds may induce apoptotic pathways at high concentrations. Nevertheless, all tested compounds exhibited low cytotoxicity at concentrations lower than 250 µg/mL and high IC_50_ values, suggesting limited cytotoxic potential (Figure 8).

**Figure 9:**
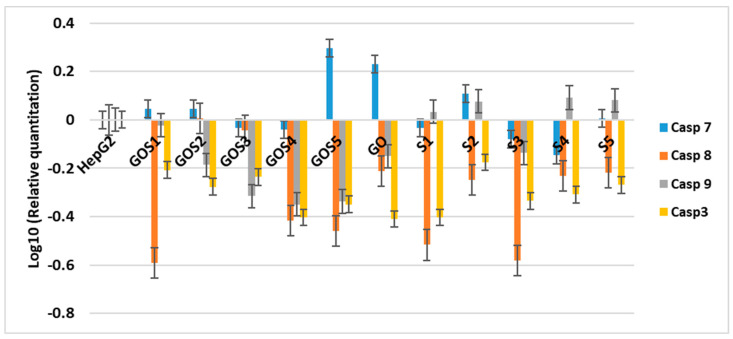
The effect of S and GO-S_x_ at 200 μg/mL on the apoptotic caspase expression.

Caspase 8, which acts in the extrinsic apoptosis pathway [38,39,40,41], was inhibited by all substances (Figure 9). Instead, the intrinsic caspase 9 [38] was increased by the sulfonamides S1, S2, S4 and S5, showing that they interfere with this apoptotic pathway. From downstream caspases, only the executioner caspase 7 was increased during the treatment with S2, GOS1, GOS2 and GOS5. Moreover, the caspase-3, which carries out the apoptosis process of the cell, was inhibited in our experiments. Taken together, these results show a weak activation of intrinsic apoptosis.

**Figure 10:**
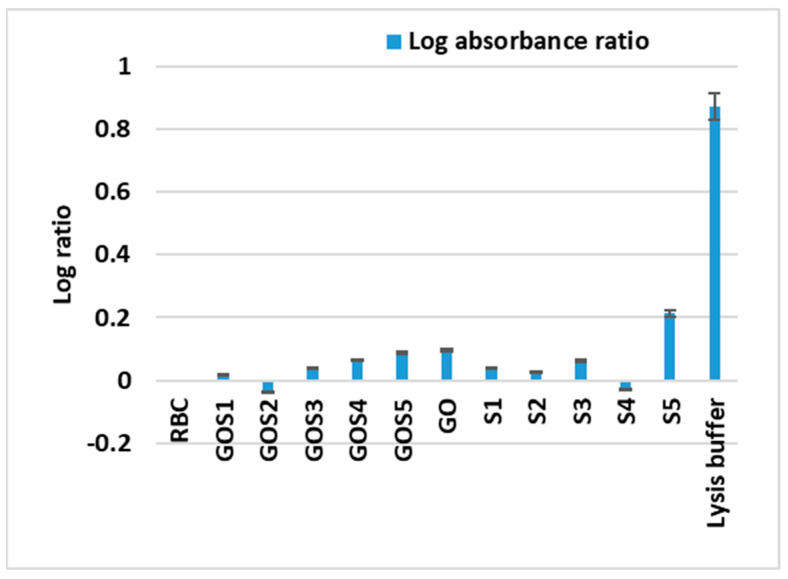
Hemotoxicity induced by S and GO-S_x_: log ratio sample/RBC absorbance.

Regarding hemolytic activity (Figure 10), all tested compounds exhibited only low blood cells toxicity in comparison with the lysis buffer used as positive control. All GO-Sx nanostructures exhibited a lower hemotoxicity as compared with the GO control. Also, excepting S4, for all the other four sulfonamides, the covalent binding to GO decreased their blood toxicity.

#### 2.2.3. Cell Cycle Analysis

**Figure 11:**
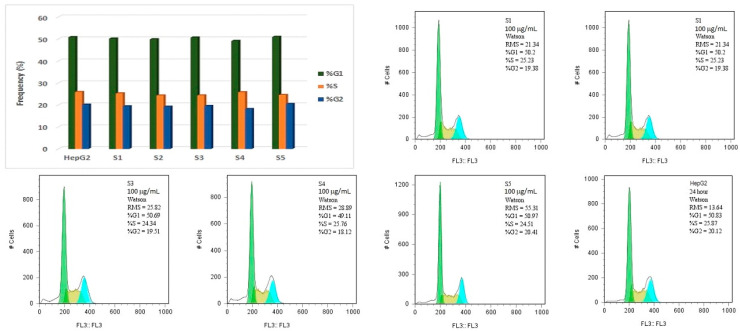
The effect of S at 100 μg/mL concentration on HepG2 cell cycle after 24 h.

**Figure 12:**
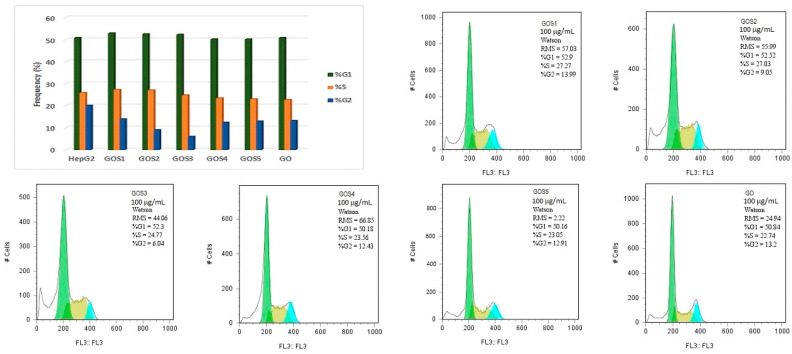
The effect of GO-S_x_ at 100 μg/mL concentration on the cell cycle of HepG2 cells after 24 h.

Cell cycle analysis of both treated and untreated HepG2 cells was conducted using flow cytometry. In untreated cells, 50.83% of the population was in the G0/G1 phase. Following 24 h treatment with 100 μg/mL of the test compounds, only minimal variations in the G0/G1 phase distribution were observed among the treated groups: 50.2% (S1), 49.87% (S2), 50.64% (S3), 49.11% (S4) and 50.97% (S5) (Figure 11). In comparison, cells treated with GO-Sx formulations exhibited a slight increase in the G0/G1 phase population: 52.9% (GOS1), 52.52% (GOS2), 52.3% (GOS3), 50.18% (GOS4) and 50.16% (GOS5) (Figure 12). Analysis of the G2/M phase revealed a marked decrease following treatment with graphene-based materials, dropping from 20.12% in untreated cells to 13.2% (GO), 13.99% (GOS1), 9.05% (GOS2), 6.04% (GOS3), 12.43% (GOS4) and 12.91% (GOS5), with the most pronounced reduction observed in the GOS3 group.

Additionally, the appearance of a sub-G0 peak in the histogram—positioned to the left of the G0/G1 peak—was noted, indicating the presence of apoptotic cells.

#### 2.2.4. Activation of Metabolism-Specific Gene Expression

**Figure 13:**
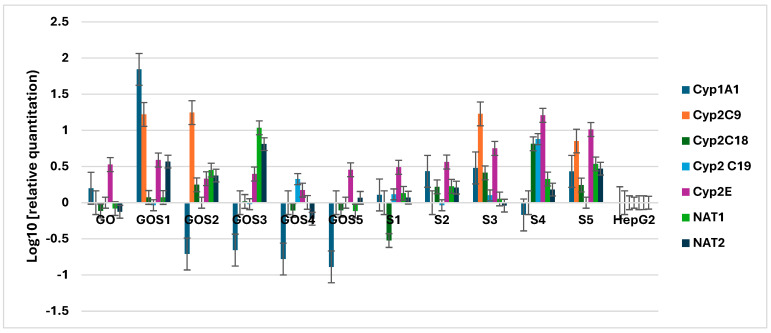
Influence of S and GO-S_x_ on the expression of antibiotic metabolism-specific genes expression.

Cxyp1A1 expression was increased in the presence of all sulfonamides excepting S4, GO and GOS1 (Figure 13). On the contrary, GOS2, GOS3, GOS4 and GOS5 induced a decrease in Cyp1A1 expression. Expression of Cyp2C9 was increased in the presence of S3, S5, GOS1 and GOS2, and of Cyp2E in the presence of all sulfonamides and their nanostructured complexes. The expression of Cyp2C18 and Cyp2C19 was basal, except for S4, that increased their expression. NAT1 and NAT2 expression was increased in the presence of S5, S4, GOS3 and GOS2.

## 3. Discussion

In our study, we aimed to evaluate the effects of some sulfonamides conjugating/or adsorption on graphene oxide on their biological activities.

We have chosen to use *para*-aminobenzene sulfonamide (sulfanilamide S1), 4-amino-*N*-(5-methyl-1,2-oxazol-3-yl) benzene sulfonamide (sulfamethoxazole S2), 4-amino-*N*-(3,4-dimethyl-1,2-oxazol-5-yl) benzene sulfonamide (sulfafurazole/sulfisoxazole, S3), 4-amino-*N*-(1,3-thiazol-2-yl) benzene sulfonamide (sulfathiazole S4). Also, we tested sulfasalazine (2-hydroxy-5-((4-(pyridin-2-ylsulfamoyl)phenyl]diazinyl)benzoic acid, S5), a sulfonamide with multiple pharmacological activities (antibacterial, anti-inflammatory and immunosuppressive). Sulfanilamide S1 (Figure 1) is a short-acting antibacterial chemotherapeutic that inhibits bacterial growth and also has carbonic anhydrase inhibitory properties. Unfortunately, its administration can cause hypersensitivity reactions [22]. Literature data shows that sulfanilamide-loaded polyvinyl alcohol–chitosan composite nanofibers and silver nanoparticles facilitate the prolonged drug release. These composite fibers, especially when fabricated by electrospinning, proved to exhibit excellent antimicrobial activity toward tested pathogenic microbes and a wound-healing activity [12]. Sulfamethoxazole S2 (Figure 1) is most commonly used in combination with trimethoprim (5-((3,4,5-trimethoxyphenyl)methyl)pyrimidine-2,4-diamine), a chemotherapeutic that inhibits dihydrofolate reductase, acquiring a bactericidal effect [23]. The combination known as co-trimoxazole has a broad spectrum of activity on *Gram-positive* and *Gram-negative* bacteria, a good tolerability profile, reduced costs and, not least, a low risk of selecting bacterial resistance [26]. In a drug repurposing strategy, it has been considered as a potential treatment for drug-resistant tuberculosis [13], as an antimalarial [14] and as a prophylactic for opportunistic infections in HIV-infected patients [15]. Sulfafurazole (sulfisoxazole) S3 (Figure 1) is a broad-spectrum sulfonamide. It has activity against a wide range of *Gram-negative* and *Gram-positive* bacteria, and it is also used in combination with phenazopyridine or erythromycin, in patients with a history of penicillin hypersensitivity or with ampicillin-resistant *Haemophilus influenzae* infections. It was demonstrated that sulfafurazole is an endothelin receptor antagonist that protects retinal neurons from insults of ischemia/reperfusion or lipopolysaccharides [20]. In drug repurposing strategies, sulfafurazole has been recently linked to anti-metastasis therapies by inhibiting the secretion of small extracellular vesicles by targeting the endothelin receptor A [21]. The main clinical problem related to sulfisoxazole is its low bioavailability [16]. Some strategies have been developed to solve its low solubility by using inclusion complexes as drug delivery systems [17,18]. Sulfathiazole S4 (Figure 1) exists in polymorph forms. The imine tautomer prevails, at least in a solid state, and the proton resides on the ring nitrogen [19]. It is used to disinfect home aquariums, in association with other drugs to treat vaginal, conjunctival or skin infections [25]. It is rarely administered systemically, because of its toxicity. Sulfasalazine S5 (Figure 1), a sulfonamide active in rheumatoid arthritis, ulcerative colitis and Crohn’s disease has an affinity for connective tissues containing elastin. It is a prodrug that is metabolized in the colon under the action of bacterial azoreductases, forming the active metabolites sulfapyridine, a local antibacterial, and mesalazine (5-aminosalicylic acid), with anti-inflammatory and immunosuppressive properties [27]. The specific mechanism of action of such sulfonamides is still not fully understood. One proposed mechanism is the inhibition of prostaglandin synthesis, resulting in local anti-inflammatory effects in the colon by inhibiting cyclooxygenase production. Also, the drug’s mechanism of action may relate to interference with arachidonic acid metabolism by the lipoxygenase pathway, modulating local chemical mediators of the inflammatory response, such as leukotrienes [42,43,44]. It is also postulated as a free radical scavenger [45]. In vitro studies for immunomodulatory effects have shown that sulfasalazine inhibits the release of proinflammatory cytokines, such as interleukin 1 (IL-1), tumor necrosis factor-alpha (TNFα), IL-2, IL-8 and NF kB [46,47]. Moreover, sulfasalazine has immunosuppressive activity by blocking the lymphocyte DNA synthesis and in vitro cell cycle progression, preventing clonal expansion of potential pathogenic T-cell and B-cell populations [48]. In vitro, sulfasalazine interferes with polymorphonuclear leukocytes and macrophage cells adhesion and, functions, including chemotaxis, phagocytosis and adhesion [49].

Despite some limitations, sulfonamides continue to be useful because they fill specific gaps in our antimicrobial arsenal, due to many reasons, such as: (i) they target the folate pathway; (ii) offer an alternative for certain organisms and patient groups; (iii) are accessible in resource-limited areas due to low cost and long history of use. Of course, prudent use is required due to resistance (acquired by alternative dihydropteroate synthase enzymes encoded by *sul1, sul2* or *sul3* genes, increased PABA production or bypass of the blocked pathway) and the risk of sulfa hypersensitivity reactions (which can be severe in a subset of patients) [8,9].

Resistance development has indeed reduced the once-broad efficacy of sulfonamides, confining their use, and has prompted research into ways to enhance the antibacterial activity of sulfonamides or restore their effectiveness using modern technologies. One promising strategy emerging in the past five years is the use of nanomaterials, such as graphene oxide, to conjugate with or deliver sulfonamide antibiotics. By leveraging nanotechnology, researchers aim to overcome some resistance mechanisms and improve drug performance (e.g., through better delivery to bacterial cells or synergistic antimicrobial actions). Graphene oxide—a single-atom-thick sheet of carbon atoms with various oxygen-containing functional groups—has gained attention as a versatile nanoplatform for drug delivery and antimicrobial therapy. In recent years, studies have explored graphene oxide–sulfonamide conjugates and nanocomposites to improve antibacterial outcomes, due to its high drug loading capacity and controlled release capacity, also demonstrated for sulfamethoxazole [50]: (i) improved delivery to target sites that can increase the local effective concentration of the sulfonamide while minimizing exposure to host tissues; (ii) good biocompatibility [51]; (iii) increased bacterial uptake and retention, by helping antibiotics overcome bacterial cell walls/membranes, due to GO adherence to bacterial surfaces and occurrence of a “wrapping” mechanism hypothesized for graphene-family materials that can isolate bacteria or concentrate antimicrobial agents at the cell surface. Additionally, GO’s sharp edges and sheet-like structure allow it to insert into or disrupt membranes (like a nanoscale blade), creating physical openings. This “nano-knife” effect can increase cell permeability, potentially letting more of the sulfonamide into the bacterial cell. In essence, GO can act as a trojan horse—carrying sulfonamide on its surface, anchoring to the microbe, perforating the cell membrane and thereby facilitating antibiotic entry where it can inhibit intracellular folate synthesis [52]; (iv) synergistic antimicrobial effects, due to oxidative stress induction mediated by the generation of reactive oxygen species (ROS) or by depleting antioxidants, leading to cellular damage or to physical damage of the bacterial membrane. These actions do not depend on the antibiotic’s mechanism, so they can attack bacteria in parallel, as already demonstrated for penicillin and oxacillin loaded on PEGylated graphene oxide that restored their ability to combat MRSA [53]. When a sulfonamide–GO conjugate is used, the bacteria are hit with a one-two punch: the sulfonamide inhibits folate/DNA synthesis biochemically, while the GO imposes mechanical and oxidative stress on the cell; (v) broad-spectrum and anti-biofilm activity: Graphene oxide is effective against a wide range of bacteria, including Gram-positive and Gram-negative species, which complements the broad spectrum of sulfonamides. In a recent experiment, glucosamine-functionalized GO loaded with sulfamethoxazole was tested against diverse bacteria—*E. coli*, *Serratia marcescens*, *Pseudomonas aeruginosa*, *Salmonella enterica* (Gram-negatives) and *Bacillus cereus*, *Streptococcus pyogenes*, *Streptococcus pneumoniae* (Gram-positives)—and the formulation showed significant antibacterial activity against all tested strains [50]. Graphene oxide can also disrupt bacterial biofilms, the protective matrices that often render infections resistant to antibiotics. By penetrating or breaking up biofilm structure (through its physical interactions and oxidative stress), GO can allow sulfonamide antibiotics to reach embedded bacteria that would otherwise be shielded. This anti-biofilm property further enhances the overall antibacterial efficacy of the sulfonamide–GO combination.

In our study, the antimicrobial activity of obtained GO-Sx was assessed against Gram-positive and Gram-negative bacteria and fungal strains, both in planktonic, but also in biofilm growth state. The MIC values of the GO-Sx were higher than those of the GO and S, demonstrating either that the pharmacologically active groups against planktonic microbes are probably chemically inactivated or blocked by steric hindrance during the covalent functionalization, or the permeability of cellular envelopes/the affinity for the microbial target decreases for the nanostructures in comparison with free antibiotics. This finding aligns with previous reports highlighting that covalent attachment can impair small molecule functionality if the bioactive groups are not spatially accessible. Nevertheless, the conjugates still exhibited meaningful activity, suggesting partial retention of antibacterial potential.

In contrast, the antibiofilm assays (MBIC values) painted a more nuanced picture. While both sulfonamides and GO–Sx conjugates showed comparable inhibition against biofilm formation, several GO-functionalized compounds—GOS3, GOS4, and GOS5—demonstrated improved antibiofilm effects compared to their non-functionalized counterparts. Notably, GOS5 exhibited the strongest inhibition of *P. aeruginosa* biofilm development, with a MBIC of 0.078 mg/mL, outperforming both free GO and free S5. This enhancement suggests that, while antibacterial efficacy against planktonic cells may be compromised upon conjugation, nanostructuring improves anti-biofilm activity, potentially through mechanisms like enhanced surface adhesion, facilitating greater uptake or localization of the drug in the biofilm matrix or even inside the bacterial, cell biofilm penetration, sustained local drug release or providing synergistic killing through its own bactericidal actions (e.g., membrane disruption, oxidative stress). These combined effects can also help sulfonamides circumvent resistance, as the bacteria are simultaneously weakened by physical/chemical stress while the drug inhibits their metabolism.

Besides investigating the antimicrobial activity of the obtained functionalized nanostructures, we have also assessed their influence on mammalian cells and processes known to be involved in drug metabolism.

The cytotoxicity evaluation using the CellTiter-Glo assay and Incucyte live-cell imaging system revealed that S and their conjugates (GO–Sx) exert low cytotoxicity toward mammalian cells at concentrations below 250 µg/mL. The high IC_50_ values further confirm the biocompatibility of these compounds in the tested dose range. Notably, only at high concentrations were morphological changes such as membrane blebbing and cell shrinkage observed—typical markers of apoptotic cell death. This suggests a dose-dependent response, with apoptotic features becoming prominent only at supra-physiological levels.

Interestingly, analysis of caspase activity provided a more nuanced insight into the apoptosis pathways involved. Caspase-8, a key mediator of the extrinsic apoptotic pathway, was inhibited by all tested substances, suggesting that these compounds do not activate apoptosis via death receptor signaling. In contrast, the upregulation of intrinsic apoptotic markers—particularly caspase-9 and, in some cases, the executioner caspase-7—by specific sulfonamides (S1, S2, S4 and S5) and several GO–Sx formulations (notably GOS1, GOS2 and GOS5) indicates partial engagement of the mitochondrial apoptotic pathway. However, the inhibition of caspase-3, a terminal executioner caspase, points to an incomplete or weak activation of apoptosis overall. This subtle pro-apoptotic signaling at higher doses should be further investigated, but the current data support a favorable toxicity profile for therapeutic applications.

The hemolytic potential of S and GO–Sx was also evaluated as a critical indicator of systemic compatibility. All tested compounds displayed low hemolytic activity compared to the positive control (lysis buffer), underscoring their potential safety in blood-contacting applications. Notably, all GO–Sx nanostructures exhibited reduced hemolytic activity relative to the GO control, suggesting that covalent functionalization with sulfonamides may mitigate the basal cytotoxicity of graphene oxide. This effect was especially evident for S1, S2, S3 and S5. The exception was S4, whose conjugation to GO did not improve hemocompatibility.

Flow cytometric analysis of HepG2 cells demonstrated minimal alterations in the G0/G1 phase population following treatment with either free sulfonamides or their GO-based conjugates at 100 µg/mL for 24 h. However, GO–Sx-treated cells displayed a slight but consistent increase in G0/G1 arrest, suggesting a mild cell cycle regulatory effect. The G2/M phase, by contrast, exhibited a substantial reduction in cell population upon exposure to GO and GO–Sx nanostructures—most notably with GOS3, which reduced the G2/M fraction from 20.12% (control) to 6.04%. This shift may indicate a blockade in cell cycle progression, potentially contributing to the antiproliferative effect of GO–Sx. Furthermore, the appearance of a sub-G0 peak, corresponding to apoptotic cell fragments, reinforces the morphological and caspase-based evidence of limited apoptosis induction, particularly in the GOS3 group.

In order to decipher the biological effects of graphene–sulfonamide compounds, we evaluated the mRNA expression of enzymes involved in drug metabolism. Both NATs (N-acetyltransferases) and CYP P450s (cytochrome P450s) are enzymes involved in the metabolism of xenobiotics (drugs, toxins, endogenous compounds) [54,55,56,57,58,59,60,61,62,63,64], but their functions and mechanisms of action are different. CYP P450 enzymes (Cytochrome P450) introduce polar groups into lipophilic molecules to make them more water-soluble, catalyze oxidation reactions (phase I metabolism), hydroxylation (-OH), epoxidation (-O-), deamination, desulfurization, etc. [65,66], are found localized in the endoplasmic reticulum and mitochondria and require NADPH and molecular oxygen for activity. CYP1A participates in the biotransformation of about 9% of clinical drugs, being the most important phase I drug metabolic enzyme [67]. NAT enzymes (N-acetyltransferases) metabolize drugs, aromatic amines and hydrazines by transferring an acetyl group (-COCH_3_) from Acetyl-CoA to substrate molecules [68]. NAT enzymes are cytoplasmic enzymes and do not depend on oxygen [69,70]. Both types of enzymes play an essential role in the detoxification of the body and can influence the efficacy of drugs and the risk of toxicity, and for this reason, we studied the activation of some of them at the mRNA level.

On the other hand, regarding graphenes, their metabolism is little studied. Graphenes and their derivatives (i.e., graphene oxide, nanographenes) are hydrophobic materials and resistant to degradation, thus difficult to be metabolized through classical enzymatic pathways and prone to tissue accumulation, leading to potential toxic effects [71]. Their insufficient clearance could also influence the pharmacokinetics of concomitantly administered drugs. Graphene metabolism has been investigated mainly through collateral studies. Thus, Valdehita et al. [72] investigated how GO influences the aryl hydrocarbon receptor (AhR) and cytochrome P4501A (Cyp1A) system activated by benzo(k)fluoranthene (BkF) in the rainbow trout cell line [73]. They showed that GO did not directly activate the AhR receptor in rainbow trout cells, but in the presence of BkF, GO potentiated the activation of the AhR-Cyp1A system, suggesting that GO may modulate the cellular response to polycyclic aromatic hydrocarbons. Thus, although the study did not directly investigate graphene metabolism, the results indicated that graphene oxide may indirectly influence cellular detoxification systems, such as AhR-Cyp1A, in the presence of other compounds, suggesting potential implications for how organisms metabolize and eliminate graphene-based nanomaterials.

On the other hand, GO was shown to induce aryl hydrocarbon receptor (AhR)-dependent activation of the cyp1a gene and promote the migration of lck+ cells in the intestine of germ-free zebrafish larvae when combined with a specific microbial consortium [74], suggesting that the interaction between GO, the gut microbiome and AhR (and cyp1a gene activation) may influence immune responses and, possibly, how the body processes and eliminates graphene-based nanomaterials.

Gene expression analysis revealed complex effects of S and GO–Sx compounds on xenobiotic metabolism-related enzymes. Cyp1A1 expression was upregulated by most sulfonamides except S4, while GO and GOS1 failed to induce this enzyme. Interestingly, GO–Sx nanostructures GOS2 to GOS5 led to a suppression of Cyp1A1, indicating a possible regulatory effect of GO conjugation on cytochrome P450 expression.

Cyp2C9 expression was enhanced by S3, S5, GOS1 and GOS2, suggesting these compounds may alter phase I metabolic processing. Similarly, Cyp2E expression was universally upregulated across all treatments, highlighting a potentially shared induction mechanism of this enzyme. On the other hand, expression of Cyp2C18 and Cyp2C19 remained largely unchanged, with the exception of S4, which markedly increased their levels—consistent with its distinct toxicity and hemolytic behavior.

Moreover, the upregulation of NAT1 and NAT2 by S4, S5, GOS2 and GOS3 indicates enhanced activity of phase II metabolic pathways. These findings suggest that the functionalization of sulfonamides with GO may modulate both phase I and phase II drug metabolism enzymes, a property that could influence their pharmacokinetics and safety profiles.

These results are sustained by other studies demonstrating that N-acetyltransferases are key enzymes for the acetylation of sulfasalazine (especially NAT-2) [75,76] and sulfamethoxazole [by acetylation of sulfamethoxazole at its N4 position].

Collectively, the results support the hypothesis that GO–Sx nanostructures possess low cytotoxicity, enhanced hemocompatibility and limited pro-apoptotic activity at sub-toxic concentrations, while retaining bioactivity. The mild cell cycle perturbation and gene expression modulation observed in HepG2 cells point to a biocompatible but biologically responsive profile, suitable for further development as antimicrobial or anticancer agents.

These findings strengthen the rationale for using graphene-based nanocarriers to improve the delivery and safety of sulfonamide derivatives. Future work should aim to elucidate the structure–activity relationship governing sulfonamide–GO interactions, assess long-term in vivo toxicity and metabolic fate and explore the therapeutic index in models of infection or tumor proliferation.

## 4. Materials and Methods

### 4.1. Materials

Graphene oxide was obtained as follows (Hummer’s method) [36,77,78,79,80,81]: 3 g of graphite and 1.5 g of sodium nitrate were added carefully under energic stirring to 75 mL of concentrated sulfuric acid. The mixture was cooled with ice and salt, and then 9 g of potassium permanganate was added in small portions. Stirring continued for 1.5 h; then, the cooling system was removed, and the solution was allowed to reach room temperature (under stirring). Then, 150 mL of cold water was added very slowly under stirring, and after 15 min, another 300 mL of cold water was added. Finally, several mL of hydrogen peroxide was added until the violet solution disappeared. The mixture was left overnight to settle without stirring, and the next day, the slurry was decantated and washed several times with hydrochloric acid, followed by methanol, and then let dry in open air. For functionalization with sulfonamides, 1 g of GO suspended in 50 mL of dichloroethane was treated with a few drops of DMF and 5 mL of thionyl chloride under reflux for 1 h (temperature about 80 °C). The solid was separated and washed with DCM, then reacted with the corresponding sulfonamide in the presence of triethylamine (1 mL). The next day, the final material was separated, washed with DCM and dried.

The mammalian cells HepG2 are traceable to the ATCC, under the accession number ATCC-HB-8065.

### 4.2. Methods for Sample Characterization

The morphological characterization of samples was performed using the SU-70 microscope (Hitachi, Ibaraki, Japan), which is a scanning electron microscope with field emission (FE-SEM) and operates under high vacuum conditions (10^−8^ Pa), allowing the acquisition of high-resolution images (1 nm resolution at 15 kV acceleration voltages). SEM investigations were performed under 10 kV accelerating voltage and 18–21 mm working distance range. The UltraDry EDS detector (Thermo Fisher Scientific, Waltham, MA, USA) coupled to an SEM microscope was used for elemental analysis under the following setup: 10 kV acceleration voltage, Phi-Rho-Z correction method available in NSS software (v. 3).

Fourier Transform Infrared Spectroscopy (FTIR) is a vibrational, nondestructive and noninvasive analytical technique that can be performed in either transmission or reflection mode. The FTIR investigations were performed with a Vertex 80v FTIR spectrometer (Bruker, Ettlingen, Germany) equipped with a diamond ATR (Attenuated Total Reflection) crystal accessory and a Hyperion 3000 microscope (Bruker, Ettlingen, Germany), within a spectral range of 400–8000 cm^−1^, a spectral resolution of 0.2 cm^−1^ and an accuracy of ±1 µm. An air background spectrum was recorded using 12 co-added scans, while the sample spectra were collected with 32 co-added scans. The signals are recorded in transmittance mode, with a high spatial resolution of just a few micrometers. FTIR imaging spectra were baseline-corrected, processed and analyzed using the Bruker OPUS v.7.5 software spectral library (Bruker, Ettlingen, Germany), under 19% relative humidity.

A Xantus-2^TM^ analyzer (Rigaku, Boston, MA, USA) with two laser sources (i.e., 785 nm and 1064 nm) and two TE cooled detectors (i.e., CCD and InGaAs) was used to record the Raman spectra. The following parameters were applied to this study: 50 mW laser power, 500 ms integration time, 5 scans/spectra and an excitation source of 1064 nm. With a spectral resolution of 15–18 cm^−1^, the spectral range was 200–2000 cm^−1^.

### 4.3. Biological Activity

#### 4.3.1. Broth Microdilution Assay

The graphene oxide (GO), graphene oxide functionalized with sulfonamides (GO-S_x_) and sulfonamides (S1–S5) were investigated for their in vitro antibacterial activity against two Gram-positive (*Staphylococcus aureus* ATCC 25923, *Enterococcus faecalis* ATCC 29212), two Gram-negative (*Escherichia coli* ATCC 25922, *Pseudomonas aeruginosa* ATCC 27853) and for antifungal activity against *Candida albicans* ATCC 10231. The evaluation of the biological activity of the compounds was performed using the broth microdilution method. The inoculum was prepared in sterile PBS (phosphate-buffered saline) by suspending four to five colonies from 18–24 h pure solid microbial cultures grown on solid media. The turbidity was adjusted to the McFarland standard 0.5 and the resulting suspensions were further diluted to 1:100 in Muller Hinton broth (MHB). A total of ten serial binary dilutions, starting from 10 mg/mL (compound stock solution in DMSO) for each tested compound and DMSO, were prepared in sterile MHB distributed in 96-well sterile microplates. Each well of the microplates containing the compound dilution was inoculated with the standard microbial suspensions. For each tested compound, a sterility control containing only broth and a growth control containing broth and microbial inoculum were prepared. The separate components, namely the sulfonamides and the GO alone, were also tested. The microplates were incubated at 37 °C for 16–24 h. The minimal inhibitory concentration (MIC) values, defined as the lowest concentrations of the compound that inhibited the microbial growth of the tested microbial strains, were determined based on the absorbance values assessed spectrophotometrically at 620 nm. The assays were performed in duplicate. 

#### 4.3.2. Crystal Violet Assay of Antibiofilm Activity

The GO, GO-S_x_ and S influence on the microbial biofilms was evaluated as previously described. Using fresh solid cultures, microbial suspensions were prepared in MHB to obtain a density of 10^7^ CFU/mL, which were further used for inoculation of 96-well plates in the presence of ten different concentrations of each compound (starting from 10 mg/mL stock solution in DMSO). Wells containing only broth served as a negative control, and wells containing broth seeded with microbial suspension as a positive control. The inoculated microplates were incubated at 37 °C for 24 h. After incubation, the liquid microbial cultures were gently discarded and rinsed three times with PBS (pH 7.2) to remove the unattached microbial cells. Then, the biofilms were fixed with 150 μL of methanol for 15 min and stained with 1% CV for 15 min at room temperature. The excess dye was washed with PBS, and 150 μL of acetic acid 33% was added to dissolve the crystal violet. The minimum biofilm inhibitory concentration (MBIC) was defined as the lowest concentration of the compound that inhibited biofilm formation, based on the values of absorbance of colored solutions at OD492 measured by using a microtiter plate spectrophotometer. The experiments were carried out in duplicate.

#### 4.3.3. Analysis of Cytotoxicity Using CellTiter 96^®^ AQueous One Solution Cell Proliferation Kit

The cells HepG2 (ATCC-HB-8065) were grown in 96-well plates at a density of 5 × 10^4^ cells/well and treated with serial dilutions of GO-S_x_ beginning with 1 mg/mL. After 24 h of treatment, 10 μL of Cell Titer 96^®^ Aqueous One Solution (Promega, Madison, WI, USA) was directly added to the culture medium and incubated for 3 h at 37 °C. The tetrazolium salt (3-(4,5-dimethylthiazol-2-yl)-5-(3-carboxymethoxyphenyl)-2-(4-sulfophenyl) -2H-tetrazolium) was reduced to a colored, water-soluble formazan product, whose concentration—quantified by measuring absorbance at 490 nm—was directly proportional to the number of viable cells. Cell growth inhibition was calculated using the following equation: Growth inhibition = (optical density of treated cells/optical density of control cells) × 100%.

#### 4.3.4. Toxicity Evaluation Using Incucyte

Cells were seeded in culture plates at 2000 cells/well and kept in the incubator for 24 h. The cells were treated with binary serial dilutions of S and GO-S_x_ at concentrations beginning with 1 mg/mL. The plates were inserted into the device, Incucyte SX1 Live Cell Analysis Instrument (Sartorius, Goettingen, Germany), for non-invasive monitoring of cell status. The analysis and interpretation of experimental results were conducted using software integrated with the Incucyte system, which facilitates real-time data processing and produces comprehensive graphical outputs and reports.

#### 4.3.5. Hemolysis Assay

Hemotoxicity was established using Lourenço’s protocol [82]. Fresh ethylenediamine tetra-acetic acid (EDTA)-stabilized human whole blood samples were obtained from a clinically healthy person. The whole blood was washed three times by dilution 1:3 in saline calcium- and magnesium-free Dulbecco’s PBS, pH 7.4 and the supernatant containing erythrocytes was collected. Two hundred microliters of erythrocyte suspension were incubated for 3 h at 37 °C with each sulfonamide and their graphene combinations at a final 200 µg/mL concentration. The experiments were performed in triplicate. ACK Lysing Buffer (Lonza, Walkersville, MD, USA) was used as a positive control of complete hemolysis. The hemolytic activity was determined by monitoring the optical density of the supernatant at 540 nm using the Tristar2S LB942 microplate multi-reader (Berthold Technologies). The hemolysis was presented as log10 of the ratio between sample absorbance and RBC absorbance.

#### 4.3.6. Cell-Cycle Analysis with Flow Cytometry

The HepG2 cells, seeded at a concentration of 10^5^ cells/cm^2^ in 24-well plates for 24 h, were treated with 100 μg/mL sulfonamides or their combinations with graphene for another 24 h and were analyzed for the cell cycle using flow cytometry. Briefly, the cells were detached by trypsin, washed with PBS and fixed in cold ethanol (70%) overnight. The cells were hydrated and incubated with 100 μL FxCycle™ PI/RNase Staining Solution (Thermo Fisher Scientific, USA) for one hour at 37 °C and an additional hour at 4 °C. Finally, the stained DNA was evaluated with an EPIX XL Beckman Coulter flow cytometer (Beckman Coulter, USA) and analyzed using FlowJo software, version 7.2.5 (USA). 

#### 4.3.7. Gene Expression Analysis by qRT-PCR

The HepG2 (3 × 10^5^ cells) were seeded in 6-well plates for 24 h and treated with 200 μg/mL sulfonamides and its combinations with graphene for another 24 h. Analysis of gene expression was performed according to Stoica et al. [83]. Briefly, RNA was isolated from treated and untreated cells using Trizol Reagent (Invitrogen, Life Technologies, Carlsbad, CA, USA) according to the manufacturer’s instructions and the total RNA was quantified using NanoDrop (Thermo Fisher Scientific, Waltham, MA, USA). cDNA synthesis was obtained from exactly 2 µg RNA by reverse transcription using a High-Capacity cDNA Reverse Transcription Kit (Thermo Fisher Scientific Inc., USA) according to the manufacturer’s protocol. The gene expression analysis was performed using Maxima SYBR Green/ROX qPCR Master Mix (Thermo Fisher Scientific Inc., USA) and Origene gene-specific primers: Casp3 (CAT#: HP207674), Casp7 (CAT#: HP233752), Casp8 (CAT#: HP234494), Casp9 (CAT#: HP205155), Cyp1A1 (CAT#: HP200471); Cyp2C8 (CAT#: HP200712); Cyp2C18 (CAT#: HP200714); Cyp2C9 (CAT#: HP200713); Cyp2C19 (CAT#: HP200711); Cyp2E1 (CAT#: HP200715); NAT1 (CAT#: HP200611); NAT2 (CAT#: 200002). The GAPDH (CAT#: HP205798) was used as an endogenous control, and the relative fold differences in gene expression were calculated based on the ∆∆Cq method section.

## 5. Conclusions

While carbon allotropes, like graphenes and graphene oxides, can exhibit toxic effects on bacteria, they can be engineered to have high biocompatibility, thus being potentially useful for the development of selective antibacterial treatments that are less harmful to human tissues. Graphene oxide can be easily functionalized with many organic or inorganic compounds, including medicines. This study presents a comprehensive evaluation of the antimicrobial, antibiofilm, cytotoxic, hemolytic and metabolic effects of five sulfonamide compounds (S1–S5) and their graphene oxide-based nanoconjugates (GO–Sx). While the free sulfonamides, especially S2, S3 and S4, demonstrated superior antimicrobial activity against planktonic bacterial and fungal strains, the GO–Sx conjugates showed enhanced antibiofilm activity, particularly against *P. aeruginosa*.

Among the tested nanoconjugates, GOS3, GOS4 and GOS5 emerged as the most promising candidates, offering a favorable balance of broad-spectrum antibiofilm efficacy, low cytotoxicity and high hemocompatibility. Although the functionalization process may dampen planktonic antimicrobial potency, it appears to provide targeted advantages in disrupting biofilm-associated infections, a critical feature for treating chronic or device-associated infections. However, it is important to note that the use of graphene and oxidized graphene in the treatment of bacterial infections is still in the research and development stage [65,66]. Future work should explore the optimization of linker chemistry to preserve pharmacophore activity post-conjugation, evaluation of the pharmacokinetics and in vivo efficacy and assessment of synergistic effects in combination therapies. Overall, GO–sulfonamide nanostructures represent a viable strategy for improving the biofilm-targeting capability of existing antibiotics, with GOS5 standing out as a leading candidate for further preclinical development targeting *P. aeruginosa* biofilm-associated infections.

## Figures and Tables

**Table 1:** Elemental composition of GO-S_x_ samples, expressed as (wt.% ± S.D.%), normalized to 100 wt.%.

Samples	EDS Elemental Composition [wt.% ± S.D.%]									
	C	N	O	Na	Al	Si	S	Cl	K	Ca
**GOS1**	53.87 ± 0.29	5.77 ± 0.39	9.93 ± 0.13	nd	0.17 ± 0.01	0.19 ± 0.02	24.55 ± 0.11	5.29 ± 0.08	0.24 ± 0.03	nd
**GOS2**	58.24 ± 0.35	6.02 ± 0.41	11.23 ± 0.12	0.24 ± 0.02	nd	nd	14.71 ± 0.55	4.63 ± 0.07	2.49 ± 0.07	2.43 ± 0.08
**GOS3**	61.10 ± 0.36	6.44 ± 0.53	6.77 ± 0.13	nd	nd	0.86 ± 0.05	15.07 ± 0.09	6.28 ± 0.13	3.47 ± 0.14	nd
**GOS4**	67.87 ± 0.33	6.99 ± 0.66	8.69 ± 0.13	nd	nd	nd	11.88 ± 0.06	4.57 ± 0.06	nd	nd
**GOS5**	61.87 ± 0.35	6.79 ± 0.59	6.44 ± 0.12	nd	0.19 ± 0.02	nd	12.92 ± 0.07	2.04 ± 0.08	2.03 ± 0.05	7.73 ± 0.15
**GO**	62.84 ± 0.29	nd	33.42 ± 0.22	nd	0.14 ± 0.01	nd	2.15 ± 0.04	0.82 ± 0.05	0.63 ± 0.03	nd

nd represents elements with not detected signal or content below the limit of detection (i.e., 0.01 wt.%).

**Table 2:** The tentative assignments of significant FTIR spectra peaks.

Wavenumber [cm^−1^] & Relative Intensity *						Vibrational Assignment
GO	GOS1	GOS2	GOS3	GOS4	GOS5	
-	-	-	3709 w	-	-	NH_2_ symmetric/asymmetric group
3680 w	-	3690 w	3687 w	-	-	NH_2_ symmetric/asymmetric group
3641 w	3646 w	3645 w	3647 w	3647 w	3645 w	NH_2_ symmetric/asymmetric group
3620 w	3624 w	3626 w	3627 w	3627 w	-	NH_2_ symmetric/asymmetric group
3408 m	3392 m	3397 m	3398 m	3392 m	3389 m	OH stretching vibration of GO
	3322 s	-	-	-	-	N-H stretching vibration of sulfonamide
-	3210 m	-	-	3208 m	-	N-H stretching vibration of sulfonamide
3169 m	3161 m	3170 m	-	-	-	C=O stretching vibration
2987 m	2978 w	2980 m	-	2985 m	-	C-H stretching vibration
-	-	-	-	-	2365 w	O=C=O stretching vibration
2350 w	2355 m	2354 m	2352 w	2354 m	2350 w	C=O stretching vibration of GO; C=C-O or O=C=O stretching vibration
-	-	-	2254 w	-	-	C-N stretching vibration
-	-	-	2201 w	-	-	C-S-N stretching vibration of sulfonamide
-	-	-	2142 w	2165 w	2171 w	C-S-N stretching vibration of sulfonamide
-	-	-	2124 w	-	2121 w	C-S-N stretching vibration of sulfonamide
-	-	-	2103 w	-	-	C-S-N stretching vibration of sulfonamide
-	-	-	2085 w	-	-	C-S-N stretching vibration of sulfonamide
-	-	-	2070 w	-	-	C-S-N stretching vibration of sulfonamide
	-	2037 w	2045 w	-	-	C-S-N stretching vibration of sulfonamide
	-	2003 w	2003 w	-	2003 w	N=C- stretching vibration
			1984 w	-	1982 w	N=C- stretching vibration
1720 m	1724 m	1714 m	1730 m	1715 m	1722 m	C=C aromatic stretch (GO and sulfonamide); C=O stretching vibration of GO
**-**	-	1694 w	1696 w	1696 w	-	N-C-O stretching vibration of sulfamethoxazole; C=O and C=C stretching vibration of GO
**-**	-	1680 w	-	-	1680 w	N-C-O stretching vibration of sulfamethoxazole; C=C stretching vibration of aromatic ring
**-**	-	1650 w	-	1644 w	1642 w	N-C-O stretching vibration of sulfamethoxazole; C=C aromatic stretch of GO
1630 s	1634 s	1622 s	-	1633 s	1632 s	C=C stretching vibration of aromatic ring of GO and sulfonamide
1616 m	1595 s	1620 m	-	1593 m	1615 m	C=C stretching vibration of aromatic ring of GO and sulfonamide
1562 w	1545 w	1555 w	1556 w	1566 w	1564 w	C=C stretching vibration (GO); C-N, N-H stretching vibration
-	1538 w	1537 w	1538 w	1539 w	1537 w	C-N, N-H stretching vibration
-	1504 m	1503 m	-	1504 w	1502 w	C-N, N-H stretching vibration
-	1474 m	1470 m	-	1471 m	1472 m	C=C aromatic stretch (GO and sulfonamide); C-H stretching vibration of amide
-	-	1453 w	1454 w	1453 w	1442 w	C-H stretching vibration
1404 m	1405 m	1408 m	1417 m	1417 m	1415 m	C-O stretching vibration (GO); N-H stretching vibration
1388 m	1394 m	1389 m	-	1394 w	1389 m	C-O stretching vibration (GO)
1357 w	1362 m	1358 w	-	-	1347 w	C-OH stretching vibration of GO; S=O stretching of –SO_2_-NH- group
-	1313 m	1312 m	-	1313 w	1326 m	C-N stretching vibration; N-H stretching vibration
1238 m	1240 m	1239 m	1243 m	1240 m	1236 m	C-O-C stretching vibration (GO)
1221 m	1217 m	1221 m	-		1229 m	C-O stretching vibration (GO)
-	1182 s	1184 s	-	1181 s	-	S=O stretch from –SO_2_-NH- group
	1142 m	1142 m	-	1132 m		S=O stretch from –SO_2_-NH- group
1085 m	1082 s	1088 m	-	1083 m	1086 m	C-O stretching vibration (GO); S=O stretch from –SO_2_-NH- group
-	903 m	-	-	-	-	S-N stretching vibration from –SO_2_-N
-	897 m	-	-	-	-	S-N stretching vibration from –SO_2_-N
-	831 w	-	-	832 w	-	C-H stretching vibration
-	675 m	-	-	667 w	-	S=O stretch from –SO_2_-NH- group
	605 m		614 w	624 w		S=O stretch from –SO_2_-NH- group
	-	-	558 w	564 w	-	C-O stretching vibration of amide group
	536 s	-	539 w	532 m	-	S=O stretch from –SO_2_-NH- group

* w-weak, m-medium, s-strong.

**Table 3:** The MICs values in mg/mL of the tested S and GO-S_x_ against the microbial strains.

Microbial Strains Tested	DMSO [%]	GO	GOS1	SS1	GOS2	SS2	GOS3	SS3	GOS4	S4	GGOS5	S5
*C. albicans* ATCC 10231	12.5	0.625	1.25	0.62	0.625	0.625	0.625	10.25	1.25	1.25	11.25	1.25
*E. coli* ATCC 25922	50	5	5	0.625	5	0.019	5	0.019	5	0.019	11.25	1.25
*E. faecalis* ATCC 29212	12.5	0.625	1.25	11.25	1.25	11.25	1.25	11.25	1.25	1.25	11.25	11.25
*P. aeruginosa* ATCC 27853	50	1.25	2.5	0.156	1.25	0.078	5	0.312	1.25	0.039	22.5	0.625
*S. aureus* ATCC 25923	50	5	5	11.25	5	0.312	5	0.078	5	0.078	55	2.5

**Table 4:** The minimum biofilm inhibition concentrations (MBICs) [mg/mL] of the tested S and GO-S_x_.

Microbial Strains Tested	DMSO [%]	GO	GOS1	S1	GOS2	S2	GOS3	S3	GOS4	S4	GOS5	S5
*C. albicans* ATCC 10231	6.25	0.31	0.625	0.625	0.625	0.625	0.625	1.25	0.625	1.25	0.625	1.25
*E. coli* ATCC 25922	12.5	1.25	1.25	1.25	1.25	0.625	1.25	0.625	1.25	0.625	1.25	1.25
*E. faecalis* ATCC 29212	12.5	1.25	1.25	2.5	1.25	1.25	1.25	1.25	1.25	1.25	1.25	1.25
*P. aeruginosa* ATCC 27853	6.25	0.625	0.625	0.312	0.625	0.156	0.625	0.312	0.31	0.078	0.625	0.625
*S. aureus* ATCC 25923	12.5	2.5	2.5	2.5	2.5	1.25	2.5	1.25	2.5	1.25	1.25	1.25

## Data Availability

Data are contained within the article.

## References

[1] Spisz P., Chylewska A., Krolicka A., Ramotowska S., Dabrowska A., Makowski M. (2021). Stimulation of Sulfonamides Antibacterial Drugs Activity as a Result of Complexation with Ru(III): Physicochemical and Biological Study. Int. J. Mol. Sci..

[2] Ovung A., Bhattacharyya J. (2021). Sulfonamide drugs: Structure, antibacterial property, toxicity, and biophysical interactions. Biophys. Rev..

[3] Asyraf P.A., Kusnadi I.F., Stefanus J., Khairinisa M.A., Abdulah R. (2022). Clinical Manifestations and Genetic Influences in Sulfonamide-Induced Hypersensitivity. Drug Healthc. Patient Saf..

[4] Goldberg E., Bishara J. (2012). Contemporary unconventional clinical use of co-trimoxazole. Clin. Microbiol. Infect..

[5] Root H., Daniels L., Marx A., Bartelt L.A., Lachiewicz A.M., van Duin D. (2021). Sulfonamides without trimethoprim in the treatment of Nocardia infections: A case report and literature review. Transpl Infect Dis..

[6] Patient Education: Methicillin-Resistant Staphylococcus aureus (MRSA) (Beyond the Basics). https://www.uptodate.com/contents/4025.

[7] Yekutiel A., Shalit I., Shadkchan Y., Osherov N. (2004). In vitro activity of caspofungin combined with sulfamethoxazole against clinical isolates of *Aspergillus* spp.. Antimicrob. Agents Chemother..

[8] Dideikin A.T., Vul’ A.Y. (2019). Graphene Oxide and Derivatives: The Place in Graphene Family. Front. Phys..

[9] Liu F.F., Zhao J., Wang S., Xing B. (2016). Adsorption of sulfonamides on reduced graphene oxides as affected by pH and dissolved organic matter. Environ. Poll..

[10] Yao Q., Fan B., Xiong Y., Jin C., Sun Q., Sheng C. (2017). 3D assembly based on 2D structure of Cellulose Nanofibril/Graphene Oxide Hybrid Aerogel for Adsorptive Removal of Antibiotics in Water. Sci. Rep..

[11] Zhuang S., Zhu X., Wang J. (2018). Kinetic, equilibrium, and thermodynamic performance of sulfonamides adsorption onto graphene. Environ. Sci. Poll. Res. Int..

[12] Ganesh M., Aziz A.S., Ubaidulla U., Hemalatha P., Saravanakumar A., Ravikumar R., Peng M.M., Choi E.Y., Jang H.T. (2016). Sulfanilamide and silver nanoparticles-loaded polyvinyl alcohol-chitosan composite electrospun nanofibers: Synthesis and evaluation on synergism in wound healing. J. Ind. Eng. Chem..

[13] Palomino J.C., Martin A. (2016). The potential role of trimethoprim-sulfamethoxazole in the treatment of drug-resistant tuberculosis. Future Microbiol..

[14] Ho J.M.W., Juurlink D.N. (2011). Considerations when prescribing trimethoprim-sulfamethoxazole. Can. Med. Assoc. J..

[15] Hernandez A.V., Thota P., Pellegrino D., Pasupuleti V., Benites-Zapata V.A., Deshpande A., Penalva Oliveira A.C., Vidal J.E. (2017). A systematic review and meta-analysis of the relative efficacy and safety of treatment regimens for HIV-associated cerebral toxoplasmosis: Is trimethoprim-sulfamethoxazole a real option?. HIV Med..

[16] Fernandez-Villa D., Aguilar M.R., Rojo L. (2019). Folic Acid Antagonists: Antimicrobial and Immunomodulating Mechanisms and Applications. Int. J. Mol. Sci..

[17] Yildiz Z.I., Celebioglu A., Uyar T. (2017). Polymer-free electrospun nanofibers from sulfobutyl ether(7)-beta-cyclodextrin (SBE(7)-beta-CD) inclusion complex with sulfisoxazole: Fast-dissolving and enhanced water-solubility of sulfisoxazole. Int. J. Pharm..

[18] Aytac Z., Sen H.S., Durgun E., Uyar T. (2015). Sulfisoxazole/cyclodextrin inclusion complex incorporated in electrospun hydroxypropyl cellulose nanofibers as drug delivery system. Colloids Surf. B Biointerfaces.

[19] Hastings J., Owen G., Dekker A., Ennis M., Kale N., Muthukrishnan V., Turner S., Swainston N., Mendes P., Steinbeck C. (2016). ChEBI in 2016: Improved services and an expanding collection of metabolites. Nucleic Acids Res..

[20] Syed H., Safa R., Chidlow G., Osborne N.N. (2006). Sulfisoxazole, an endothelin receptor antagonist, protects retinal neurones from insults of ischemia/reperfusion or lipopolysaccharide. Neurochem. Int..

[21] Im E.J., Lee C.H., Moon P.G., Rangaswamy G.G., Lee B., Lee J.M., Lee J.C., Jee J.G., Bae J.S., Kwon T.K. (2019). Sulfisoxazole inhibits the secretion of small extracellular vesicles by targeting the endothelin receptor A. Nat. Commun..

[22] National Center for Biotechnology Information PubChem Compound Summary for CID 5333, Sulfanilamide. https://pubchem.ncbi.nlm.nih.gov/compound/Sulfanilamide.

[23] National Center for Biotechnology Information PubChem Compound Summary for CID 5329, Sulfamethoxazole. https://pubchem.ncbi.nlm.nih.gov/compound/Sulfamethoxazole.

[24] Kemnic T.R., Coleman M. (2022). Trimethoprim Sulfamethoxazole.

[25] National Center for Biotechnology Information PubChem Compound Summary for CID 5340, Sulfathiazole. https://pubchem.ncbi.nlm.nih.gov/compound/Sulfathiazole.

[26] Visentin M., Zhao R., Goldman I.D. (2012). The antifolates. Hematol. Oncol. Clin. North. Am..

[27] National Center for Biotechnology Information PubChem Compound Summary for 5339, Sulfasalazine. https://pubchem.ncbi.nlm.nih.gov/compound/Sulfasalazine.

[28] Kumar N., Srivastava V.C. (2018). Simple Synthesis of Large Graphene Oxide Sheets via Electrochemical Method Coupled with Oxidation Process. ACS Omega.

[29] Marcano D.C., Kosynkin D.V., Berlin J.M., Sinitskii A., Sun Z., Slesarev A., Alemany L.B., Lu W., Tour J.M. (2010). Improved Synthesis of Graphene Oxide. ACS Nano.

[30] Shahriary L., Athawale A.A. (2014). Graphene Oxide Synthesized by Using Modified Hummers Approach. Int. J. Renew. Energy Environ. Eng..

[31] Guerrero-Contreras J., Caballero-Briones F. (2015). Graphene oxide powders with different oxidation degree, prepared by synthesis variations of the Hummers method. Mater. Chem. Phys..

[32] King A.A.K., Davies B.R., Noorbehesht N., Newman P., Church T.L., Harris A.T., Raza J.M., Minett A.I. (2016). A New Raman Metric for the Characterisation of Graphene oxide and its Derivatives. Sci. Rep..

[33] Verma S., Dutta R.K. (2017). Development of Cysteine Amide Reduced Graphene Oxide (CARGO) Nano-Adsorbents for Enhanced Uranyl Ions Removal from Aqueous Medium. J. Environ. Chem. Eng..

[34] Negrea A., Bacinschi Z., Bucurica I.A., Teodorescu S., Stirbescu R.M. (2016). A New material for bipolar plates used in fuel cells. Rom. J. Phys..

[35] Kurniasari K., Maulana A., Nugraheni A.Y., Jayanti D.N., Mustofa S., Baqiya M.A., Darminto D. (2017). Defect and Magnetic Properties of Reduced Graphene Oxide Prepared from Old Coconut Shell. IOP Conf. Ser. Mater. Sci. Eng..

[36] Hummers W.S., Offeman R.E. (1958). Preparation of Graphitic Oxide. J. Am. Chem. Soc..

[37] Childres I., Jauregui L.A., Park W., Cao H., Chen Y.P., Jang J.I. (2013). Raman Spectroscopy of Graphene and Related Materials. New Developments in Photon and Materials Research.

[38] Kominami K., Nakabayashi J., Nagai T., Tsujimura Y., Chiba K., Kimura H., Miyawaki A., Sawasaki T., Yokota H., Manabe N. (2012). The molecular mechanism of apoptosis upon caspase-8 activation: Quantitative experimental validation of a mathematical model. Biochim. Biophys. Acta.

[39] Li P., Zhou L., Zhao T., Liu X., Zhang P., Liu Y., Zheng X., Li Q. (2017). Caspase-9: Structure, mechanisms and clinical application. Oncotarget.

[40] Toplicanin A., Toncev L., Matovic Zaric V., Sokic Milutinovic A. (2022). Autoimmune Hemolytic Anemia in Inflammatory Bowel Disease-Report of a Case and Review of the Literature. Life.

[41] Teplitsky V., Virag I., Halabe A. (2000). Immune complex haemolytic anaemia associated with sulfasalazine. BMJ.

[42] Peppercorn M.A. (1984). Sulfasalazine. Pharmacology, clinical use, toxicity, and related new drug development. Ann. Intern. Med..

[43] Plosker G.L., Croom K.F. (2005). Sulfasalazine: A review of its use in the management of rheumatoid arthritis. Drugs.

[44] Miller D.K., Gillard J.W., Vickers P.J., Sadowski S., Leveille C., Mancini J.A., Charleson P., Dixon R.A., Ford-Hutchinson A.W., Fortin R. (1990). Identification and isolation of a membrane protein necessary for leukotriene production. Nature.

[45] Ahnfelt-Ronne I., Nielsen O.H., Christensen A., Langholz E., Binder V., Riis P. (1990). Clinical evidence supporting the radical scavenger mechanism of 5-aminosalicylic acid. Gastroenterology.

[46] Neurath M.F. (2014). Cytokines in inflammatory bowel disease. Nat. Rev. Immunol..

[47] Neurath M.F. (2024). Strategies for targeting cytokines in inflammatory bowel disease. Nat. Rev. Immunol..

[48] Stevens C., Lipman M., Fabry S., Moscovitch-Lopatin M., Almawi W., Keresztes S., Peppercorn M.A., Strom T.B. (1995). 5-Aminosalicylic acid abrogates T-cell proliferation by blocking interleukin-2 production in peripheral blood mononuclear cells. J. Pharmacol. Exp. Ther..

[49] Neal T.M., Winterbourn C.C., Vissers M.C. (1987). Inhibition of neutrophil degranulation and superoxide production by sulfasalazine. Comparison with 5-aminosalicylic acid, sulfapyridine and olsalazine. Biochem. Pharmacol..

[50] Jabri T., Khan N.A., Makhlouf Z., Akbar N., Gul J., Shah M.R., Siddiqui R. (2023). Antibacterial Properties of Ethacridine Lactate and Sulfmethoxazole Loaded Functionalized Graphene Oxide Nanocomposites. Antibiotics.

[51] AbouAitah K., Sabbagh F., Kim B.S. (2023). Graphene Oxide Nanostructures as Nanoplatforms for Delivering Natural Therapeutic Agents: Applications in Cancer Treatment, Bacterial Infections, and Bone Regeneration Medicine. Nanomaterials.

[52] Gungordu Er S., Edirisinghe M., Tabish T.A. (2023). Graphene-Based Nanocomposites as Antibacterial, Antiviral and Antifungal Agents. Adv. Healthc. Mater..

[53] Mohammadi Tabar M., Khaleghi M., Bidram E., Zarepour A., Zarrabi A. (2022). Penicillin and Oxacillin Loaded on PEGylated-Graphene Oxide to Enhance the Activity of the Antibiotics against Methicillin-Resistant *Staphylococcus aureus*. Pharmaceutics.

[54] Brentnall M., Rodriguez-Menocal L., De Guevara R.L., Cepero E., Boise L.H. (2013). Caspase-9, caspase-3 and caspase-7 have distinct roles during intrinsic apoptosis. BMC Cell Biol..

[55] Elzagallaai A.A., Sultan E.A., Bend J.R., Abuzgaia A.M., Loubani E., Rieder M.J. (2020). Role of Oxidative Stress in Hypersensitivity Reactions to Sulfonamides. J. Clin. Pharmacol..

[56] Ou L., Song B., Liang H., Liu J., Feng X., Deng B., Sun T., Shao L. (2016). Toxicity of graphene-family nanoparticles: A general review of the origins and mechanisms. Part. Fibre Toxicol..

[57] Guo X., Mei N. (2014). Assessment of the toxic potential of graphene family nanomaterials. J. Food Drug Anal..

[58] Wadhwa R., Aggarwal T., Thapliyal N., Kumar A., Priya, Yadav P., Kumari V., Charan Reddy B.S., Chandra P., Maurya P.K. (2019). Red blood cells as an efficient in vitro model for evaluating the efficacy of metallic nanoparticles. 3 Biotech..

[59] Bissinger R., Bhuyan A.A.M., Qadri S.M., Lang F. (2019). Oxidative stress, eryptosis and anemia: A pivotal mechanistic nexus in systemic diseases. FEBS J..

[60] de Sousa Maia D.L., Côa F., da Silva K.B., Martins C.H.Z., Franqui L.S., Fonseca L.C., da Silva D.S., de Souza Delite F., Martinez D.S.T., Alves O.L. (2024). Drying of graphene oxide: Effects on red blood cells and protein corona formation. J. Mater. Sci..

[61] Kenry (2018). Understanding the hemotoxicity of graphene nanomaterials through their interactions with blood proteins cells. J. Mater. Res..

[62] Palmieri V., Perini G., De Spirito M., Papi M. (2019). Graphene oxide touches blood: In vivo interactions of bio-coronated 2D materials. Nanoscale Horiz..

[63] Taneva S.G., Krumova S., Bogar F., Kincses A., Stoichev S., Todinova S., Danailova A., Horvath J., Nasztor Z., Kelement L. (2021). Insights into graphene oxide interaction with human serum albumin in isolated state and in blood plasma. Int. J. Biol. Macromol..

[64] Ding Z., Ma H., Chen Y. (2014). Interaction of graphene oxide with human serum albumin and its mechanism. RSC Adv..

[65] Esteves F., Rueff J., Kranendonk M. (2021). The Central Role of Cytochrome P450 in Xenobiotic Metabolism-A Brief Review on a Fascinating Enzyme Family. J. Xenobiot..

[66] Sim E., Abuhammad A., Ryan A. (2014). Arylamine N-acetyltransferases: From drug metabolism and pharmacogenetics to drug discovery. Br. J. Pharmacol..

[67] Phang-Lyn S., Llerena V.A. (2022). Biochemistry, Biotransformation.

[68] Iacopetta D., Ceramella J., Catalano A., Scali E., Scumaci D., Pellegrino M., Aquaro S., Saturnino C., Sinicropi M.S. (2023). Impact of Cytochrome P450 Enzymes on the Phase I Metabolism of Drugs. Appl. Sci..

[69] Lu J., Shang X., Zhong W., Xu Y., Shi R., Wang X. (2020). New insights of CYP1A in endogenous metabolism: A focus on single nucleotide polymorphisms and diseases. Acta Pharm. Sin. B.

[70] Sim E., Fakis G., Laurieri N., Boukouvala S. (2012). Arylamine N-acetyltransferases—from drug metabolism and pharmacogenetics to identification of novel targets for pharmacological intervention. Adv. Pharmacol..

[71] Tabish T.A., Pranjol M.Z.I., Jabeen F., Abdullah T., Latif A., Khalid A., Ali M., Hayat H., Winyard P.G., Whatmore J.L. (2018). Investigation into the toxic effects of graphene nanopores on lung cancer cells and biological tissues. Appl. Mater. Today.

[72] Valdehita A., Fernandez-Cruz M.L., Navas J.M. (2023). The Potentiating Effect of Graphene Oxide on the Arylhydrocarbon Receptor (AhR)-Cytochrome P4501A (Cyp1A) System Activated by Benzo(k)fluoranthene (BkF) in Rainbow Trout Cell Line. Nanomaterials..

[73] Peng G., Sinkko H.M., Alenius H., Lozano N., Kostarelos K., Brautigam L., Fadeel B. (2023). Graphene oxide elicits microbiome-dependent type 2 immune responses via the aryl hydrocarbon receptor. Nat. Nanotechnol..

[74] Chen M., Xia B., Chen B., Guo Q., Li J., Ye M., Hu Z. (2007). N-acetyltransferase 2 slow acetylator genotype associated with adverse effects of sulphasalazine in the treatment of inflammatory bowel disease. Can. J. Gastroenterol..

[75] Tanaka E., Taniguchi A., Urano W., Nakajima H., Matsuda Y., Kitamura Y., Saito M., Yamanaka H., Saito T., Kamatani N. (2002). Adverse effects of sulfasalazine in patients with rheumatoid arthritis are associated with diplotype configuration at the N-acetyltransferase 2 gene. J. Rheumatol..

[76] Cribb A.E., Nakamura H., Grant D.M., Miller M.A., Spielberg S.P. (1993). Role of polymorphic and monomorphic human arylamine N-acetyltransferases in determining sulfamethoxazole metabolism. Biochem. Pharmacol..

[77] Avramescu S., Petrescu S., Culita D.C., Tudose M., Hanganu A., Zarafu I., Ionita P. (2020). A mixed organic functionalized silica-graphene oxide as advanced material for pollutant removal. J. Nanopart Res..

[78] Zarafu I., Turcu I., Culita D.C., Petrescu S., Popa M., Chifiriuc M.C., Limban C., Telehoiu A., Ionita P. (2018). Antimicrobial Features of Organic Functionalized Graphene-Oxide with Selected Amines. Materials.

[79] Zarafu I., Limban C., Radulescu C., Dulama I.D., Nuta D.C., Chirita C., Chifiriuc M.C., Badiceanu C.D., Popa M., Bleotu C. (2022). Novel Structures of Functionalized Graphene Oxide with Hydrazide: Characterization and Bioevaluation of Antimicrobial and Cytocompatibility Features. Coatings.

[80] Mük G.R., Popa M., Chifiriuc M.C., Voicu S.N., Florea M., Neatu F., Mihalache I., Anghel E.M., Culita D.C., Mitran R.A. (2023). Aminocoumarin Derivatives Grafted on Graphene Oxide—New Antimicrobial Agents to Combat the Resistance of Mycobacterium Tuberculosis and Eskape Pathogens. Appl. Surf. Sci..

[81] Tudose M., Anghel E.M., Culita D.C., Somacescu S., Calderon-Moreno J., Tecuceanu V., Dumitrascu F.D., Dracea O., Popa M., Marutescu L. (2021). Covalent coupling of tuberculostatic agents and graphene oxide: A promising approach for enhancing and extending their antimicrobial applications. Appl. Surf. Sci..

[82] Lourenco A.L., Saito M.S., Dorneles L.E., Viana G.M., Sathler P.C., Aguiar L.C., de Padula M., Souza Domingos T.F., Manssour Fraga A.G., Rodrigues C.R. (2015). Synthesis and antiplatelet activity of antithrombotic thiourea compounds: Biological and structure-activity relationship studies. Molecules.

[83] Stoica S.I., Onose G., Pitica I.M., Neagu A.I., Ion G., Matei L., Dragu L.D., Radu L.E., Chivu-Economescu M., Necula L.G. (2023). Molecular Aspects of Hypoxic Stress Effects in Chronic Ethanol Exposure of Neuronal Cells. Curr. Issues Mol. Biol..

